# Association of Subclinical Hypothyroidism with Type 2 Diabetes Mellitus in Qatar: A Cross-Sectional Study

**DOI:** 10.2147/DMSO.S428987

**Published:** 2023-10-28

**Authors:** AlMaha Fakhroo, Mohamed Ragab Elhadary, Basel Elsayed, Alreem Al-Kuwari, Roaa Aly, Rowan Mesilhy, Amena Bakalaf, Mazyona Al-Maadhadi, Albandare A Al-Dehaimi, Tawanda Chivese, Giridhara Rathnaiah Babu

**Affiliations:** 1 College of Medicine, QU Health, Qatar University, Doha, 2713, Qatar; 2 Department of Population Medicine, College of Medicine, QU Health, Qatar University, Doha, 2713, Qatar

**Keywords:** cross-sectional, diabetes, hypothyroidism, subclinical hypothyroidism, T2DM

## Abstract

**Purpose:**

The relationship between subclinical hypothyroidism and type 2 diabetes mellitus (T2DM) in Qatar is under-studied, despite the high prevalence of diabetes in the region. This study evaluates the potential association between subclinical hypothyroidism and T2DM in Qatar.

**Patients and Methods:**

A cross-sectional study used participants with and without T2DM from the Qatar Biobank (QBB). Logistic regression analysis was used to assess the association between subclinical hypothyroidism and T2DM, with multivariable logistic regression used to adjust for potential confounders.

**Results:**

The study found that subclinical hypothyroidism was significantly associated with a 2.82 increase in the odds of having T2DM (OR=2.82, 95% CI (1.13, 7.02), p=0.026) after adjusting for potential confounders. The proportion of subclinical hypothyroidism among individuals with T2DM in Qatar was 4.6%, significantly higher than in those without T2DM (2.8%, p=0.18).

**Conclusion:**

This study demonstrates a significant association between subclinical hypothyroidism and T2DM in Qatar. Further research is required to investigate the directionality of this association and its clinical implications.

## Introduction

Diabetes mellitus represents one of the most prevalent endocrinopathies globally.^1^ The pathogenesis of type 2 diabetes mellitus (T2DM) involves multifactorial elements, including impaired insulin sensitivity in target tissues, which leads to glucolipotoxicity, beta-cell dysfunction, and inadequate insulin secretion.^2^ Factors contributing to insulin resistance encompass obesity, overweight status, sedentary lifestyles, genetic predisposition, and aging. Emerging evidence underscores the intricate interplay between environmental factors and molecular pathways in the malfunctioning of beta-cells in T2DM.^3^ Obesity, for instance, is linked to glucotoxicity and lipotoxicity, culminating in oxidative stress and inflammation, ultimately inflicting damage upon beta-cells.^4^ The hallmark clinical manifestations of T2DM, characterized by polyuria, polydipsia, and fatigue, result from a vicious cycle of insulin resistance, hyperglycemia, and beta-cell dysfunction. The International Diabetes Federation (IDF) estimated the global prevalence of diabetes mellitus among individuals aged 20–79 to be 10.5% (536.6 million) in 2021.^5^ However, within the population of Qatari adults aged 20–79, the prevalence was notably higher, reaching 16.4% in 2020.^6^ Furthermore, projections indicate that the prevalence of T2DM in Qatar may rise to at least 24.0% by 2050.^7^

Concurrently, hypothyroidism represents another common endocrinopathy characterized by the underactivity of the thyroid gland, resulting in an insufficiency of thyroid hormones triiodothyronine (T3) and thyroxine (T4). The prevalence of hypothyroidism spans a range of 0.2–5.3% in the general population of Europe and 0.3–3.7% in the USA.^8^ In Qatar, the prevalence of hypothyroidism was reported to be 4.74% in 2021.^9^ However, a comprehensive review of thyroid disorders in the Arab world conducted in 2016 unveiled a diverse prevalence, ranging from 6.18% in Libya to 47.34% in Makkah, Saudi Arabia.^10^ Hypothyroidism can be categorized based on thyroid hormones and thyroid-stimulating hormone (TSH) levels, encompassing primary hypothyroidism (including overt and subclinical forms) and central hypothyroidism. Overt hypothyroidism is characterized by low free T4 (fT4) levels despite elevated TSH levels. Subclinical hypothyroidism, marked by normal fT4 levels and an elevated TSH, is another variant. Additionally, central hypothyroidism can be diagnosed when fT4 levels are low without an appropriate rise in TSH.^11^

The literature exploring the relationship between thyroid dysfunction and T2DM presents inconsistent findings, highlighting bidirectionality as both an antecedent and a consequence.^12^ T2DM has the potential to disrupt the response of TSH to thyrotropin-releasing hormone (TRH), culminating in hypothyroidism and reduced T3 levels.^13^ In addition, impairments in the peripheral conversion of T4 to T3 may also contribute to low T3 levels in individuals with poorly controlled diabetes.^14^ A cross-sectional study revealed a substantial prevalence of hypothyroidism (20%) among participants with T2DM.^15^ Moreover, a meta-analysis of 12 prospective studies underscored the association between abnormal thyroid hormone levels and an elevated risk of T2DM.^13^ Given the disparate findings in the existing literature, there is a compelling need for further investigation.

Despite the high prevalence of T2DM in Qatar, a comprehensive study exploring the relationship between T2DM and thyroid dysfunction remains conspicuously absent. Therefore, our study endeavors to investigate whether an association exists between subclinical hypothyroidism and T2DM within the Qatar BioBank, bridging this critical gap in knowledge.

## Materials and Methods

### Study Design

We conducted a cross-sectional study analysed as a case control using Qatar Biobank (QBB) data. The QBB database comprises Qatari citizens and long-term residents who voluntarily agreed to be part of the study.

### Study Population and Sampling Strategy

Cases and controls were selected randomly from adults aged between 18–85 years. Cases included 285 participants with T2DM, identified using one or more of the following criteria: fasting blood sugar (FBS) greater than or equal to 7 mmol/L, or random blood sugar (RBS) greater than or equal to 11 mmol/L, or HbA1c greater than or equal to 6.5%, or medical history of T2DM, based on the answer to the question “has a doctor ever told you that you had or have diabetes”? Controls were 637 participants without T2DM, identified as those with FBS less than 7 mmol/L, RBS less than 11.1 mmol/L, HbA1c less than 6.5%, and no medical history of T2DM. Participants with subclinical hypothyroidism were identified as having a TSH concentration greater than 4.5 mIU/L and normal free T4 (within 10.3 to 23.2 pmol/L), while participants without subclinical hypothyroidism had normal thyroid function tests.

Socio-demographic and other data of interest collected from the QBB included age, gender, nationality, Waist-Hip ratio (WHR), education, employment, and smoking status. Age was divided into age groups; 18–29, 30–39, 40–49, and 50–85 years. Nationality was grouped into Qatari and Non-Qatari subjects. The level of education was divided into high school or lower and undergraduate (UG) or postgraduate (PG). WHR levels were divided into two categories: normal and high. Employment status was divided into unemployed and employed or retired. Smoking habits were denoted as non-smoker, smoker, and ex-smoker.

### Statistical Analysis

The data were examined for duplicate, missing, or incomplete entries. The categorical variables were presented as frequencies (n) and percentages (%), while normally distributed numerical variables were expressed as mean and standard deviation. Skewed numerical variables were expressed as the median and interquartile range (IQR). Histograms were used to examine the normality of distribution for continuous variables. When appropriate, the chi-squared test was used to check the association between different categorical variables. In contrast, Fisher’s exact test was used for variables that did not fulfill the assumptions for the Chi-squared test.

A directed acyclic graph (DAG) was constructed based on a literature review to identify confounders, which were then adjusted for in the models (Supplementary Figure 1). Confounders identified from the DAG included: smoking, age, and gender. We also adjusted for socioeconomic status and WHR, as they were important prognostic variables for T2DM.

The association between subclinical hypothyroidism and T2DM was assessed, first using a simple logistic regression and then followed by a multivariable logistic regression, which was adjusted for possible confounders. The analysis was done using Stata software version 17. We reported the odds ratio (OR) with a 95% confidence interval (CI) for all the analyses. We reported exact P-values.

### Ethical Statement

This study was approved by QBB with the reference number QF-QBB-RES-ACC-00133. In addition, the research had received ethics approval from IRB with an approval number QU-IRB1228-E/20. At the QBB, participants gave written informed consent before participating in the study.

## Results

A total of 1000 observations were considered for analysis. The final analysis included 922 participants, as 78 participants did not have data on TSH levels, T2DM, some variables like smoking, employment, and education, or they had T1DM. Of these 922 participants, 285 had T2DM, and 637 did not.

**Figure 1:**
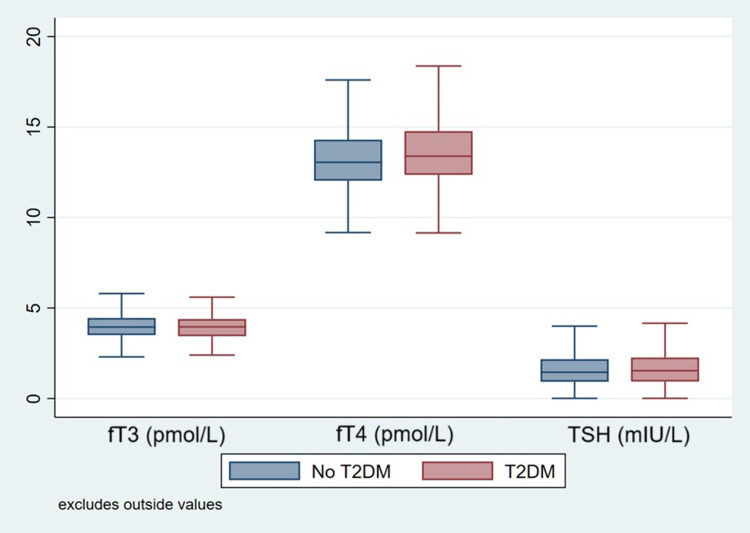
Thyroid-stimulating hormone (TSH), free triiodothyronine (fT3), and free thyroxine (fT4) levels in subjects with and without T2DM.

Table 1 summarizes the demographic characteristics in addition to HbA1C and thyroid function tests of the analysed sample. Females constituted 56.8% of the study participants. In the overall sample, 786 (85.2%) were Qataris, and 136 (14.8%) were non-Qataris (p=0.99). The mean age among participants with T2DM was 52.4 years old, while the mean age in those without T2DM was 37.0 years old. Moreover, 60.0% of participants with T2DM were 50–85 years old, compared to 14.6% of those without T2DM in the same category (P<0.001). Most participants with T2DM had a WHR of 63.5% compared to 22.6% of those without T2DM. On the other hand, the percentage of normal WHR was higher in participants without T2DM, 77.4%, compared to those with T2DM, 36.5% (P<0.001). In both groups, the largest number of participants were non-smokers, with 76.5% in the T2DM group and 71.7% in those without T2DM (p=0.014). The median values of TSH, fT4, and fT3 were almost similar across both groups, as shown in Figure 1.

**Table 1:** Characteristics of All Participants

Variable	Level	Without T2DM, N= 637	T2DM, N=285	P-value
Age (years)	18–29	178 (27.9%)	6 (2.1%)	<0.001
	30–39	227 (35.6%)	31 (10.9%)	
	40–49	139 (21.8%)	77 (27.0%)	
	50–85	93 (14.6%)	171 (60.0%)	
Gender	Female, n(%)	357 (56.0%)	166 (58.2%)	0.53
	Male, n(%)	280 (44.0%)	119 (41.8%)	
Nationality	Non-Qatari, n(%)	94 (14.8%)	42 (14.7%)	0.99
	Qatari, n(%)	543 (85.2%)	243 (85.3%)	
Education	Highschool or lower, n(%)	314 (49.3%)	193 (67.7%)	<0.001
	UG/PG, n(%)	323 (50.7%)	92 (32.3%)	
Waist-Hip ratio	Normal	493 (77.4%)	104 (36.5%)	<0.001
	High	144 (22.6%)	181 (63.5%)	
Employment	Unemployed	168 (26.4%)	85 (29.8%)	0.28
	Employed/Retired	469 (73.6%)	200 (70.2%)	
Smoking	Non-smoker	457 (71.7%)	218 (76.5%)	0.014
	Smoker	133 (20.9%)	38 (13.3%)	
	Ex-smoker	47 (7.4%)	29 (10.2%)	
Thyroid stimulating hormone (mIU/L), median (IQR)	-	1.4 (0.9, 2.2)	1.5 (0.9, 2.3)	0.31
Free Thyroxine (pmol/L), median (IQR)	-	13.1 (12.0, 14.3)	13.4 (12.4, 14.8)	0.004
Free Triiodothyronine (pmol/L), median (IQR)	-	4.0 (0.7)	3.9 (0.7)	0.29
HbA1C%, median (IQR)	-	5.2 (5.0, 5.4)	7.0 (6.1, 8.1)	<0.001

**Figure 2:**
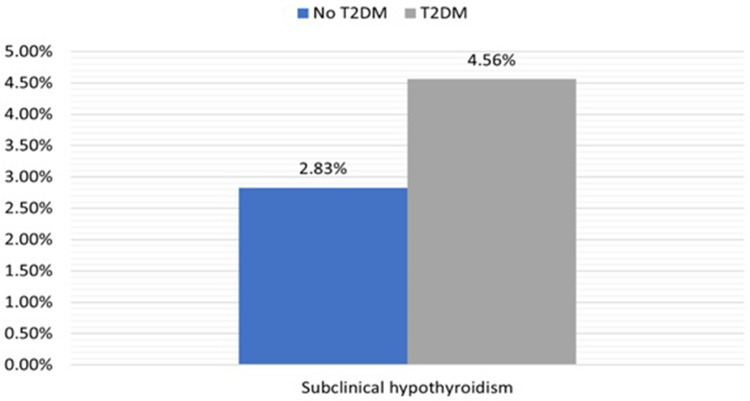
Proportions of participants with subclinical hypothyroidism in subjects with and without T2DM.

Table 2 shows the distribution of subclinical hypothyroidism among participants with and without T2DM. Overall, 31 participants had subclinical hypothyroidism (T2DM: 13 (4.6%), without T2DM: 18 (2.8%), p=0.18). Figure 2 shows the proportions of participants with subclinical hypothyroidism in those with and without T2DM.

**Table 2:** Proportions of Subclinical Hypothyroidism in Subjects with and without T2DM

Variable	Level	Without T2DM, N= 637	T2DM, N=285	P-value
Subclinical hypothyroidism	No, n(%)	619 (97.2%)	272 (95.4%)	0.18
	Yes, n(%)	18 (2.8%)	13 (4.6%)

Our results show 64% higher odds of having T2DM in participants with subclinical hypothyroidism (OR=1.64, 95% CI (0.79, 3.40), p=0.181) (Table 3). After adjusting for covariates, there was an increase in the odds of having T2DM in participants with subclinical hypothyroidism (OR=2.82, 95% CI (1.13, 7.02), p=0.026). The age category 50–85 was associated with the highest increase in the odds of having T2DM (OR=29.52, 95% CI (12.27, 71.03), p=0.000). High waist-hip ratio (WHR) was associated with increased odds of having T2DM (OR=3.54, 95% CI (2.40, 5.22), p=0.000), with strong evidence against the model hypotheses (Table 3). The baseline odds for both models are approximately 0.04. The odds ratios for the remaining variables in the multivariable logistic regressions are presented in Supplementary Table 1.

**Table 3:** Multivariable Logistic Regression for the Association Between Subclinical Hypothyroidism and Diabetes

Exposure	Levels	Controls, n(%)	Cases, n(%)	Unadjusted			Adjusted^a^			Constant^b^
				OR	95% CI	P-value	OR	95% CI	p-value	
Subclinical Hypothyroidism	No	619 (97.2%)	272 (95.4%)	Ref			Ref			0.04 (0.02, 0.09)
	Yes	18 (2.8%)	13 (4.6%)	1.64	(0.79, 3.40)	0.181	2.82	(1.13, 7.02)	0.026	
Age (years)	18–29	178 (27.9%)	6 (2.1%)	Ref						
	30–39	227 (35.6%)	31 (10.9%)	4.05	(1.65, 9.92)	0.002	3.22	(1.29, 8.02)	0.012	
	40–49	139 (21.8%)	77 (27.0%)	16.43	(6.96, 38.83)	0.000	11.68	(4.84, 28.21)	0.000	
	50–85	93 (14.6%)	171 (60.0%)	54.55	(23.27, 127.86)	0.000	29.52	(12.27, 71.03)	0.000	
Waist-Hip ratio (WHR)	Normal	493 (77.4%)	104 (36.5%)	Ref						
	High	144 (22.6%)	181 (63.5%)	5.96	(4.39, 8.08)	0.000	3.54	(2.40, 5.22)	0.000	

**Notes**: ^a^Multivariable logistic regression models adjusted for age, gender, education, waist-hip ratio, employment, and smoking. ^b^Constant reflects baseline odds, not odds ratio.

Regarding the logistic regression diagnostics, the multivariable logistic regression model fitted for this sample showed adequate goodness-of-fit with an area under the receiver operating characteristic (ROC) curve of 0.85 for the adjusted regression model (Supplementary Figure 2), and the link test showed that the model was correctly fitted and without specification errors.

## Discussion

In this cross-sectional study of adults in Qatar, we found that subclinical hypothyroidism was strongly associated with an almost three-fold increase in the odds of having T2DM. In addition, the age category 50–85 was associated with an almost 30-fold increase in the odds of having T2DM, and the high WHR was associated with an almost 4-fold increase in the odds of having T2DM.

Our findings concur with other published evidence linking subclinical hypothyroidism to T2DM. Evidence of the co-existence of both diseases ranges from 7.9% to 23%.^15^ A systematic review and meta-analysis of cross-sectional studies have established that T2DM was associated with a 1.93-fold increase in the odds of subclinical hypothyroidism.^16^ The directionality of this association remains unclear from the published literature, suggesting a putative role in both directions. One of the proposed mechanisms suggests that changes in serum TSH and fT4 levels correlate with changes in HbA1C and result in increased insulin resistance, ultimately leading to T2DM.^17^,^18^ For example, T2DM reduces the TSH levels, which impairs the conversion of T4 to T3 in the peripheral tissues, leading to subclinical hypothyroidism.^19^ On the other hand, another study suggested that an increase in serum TSH characterizes subclinical hypothyroidism, and those TSH molecules bind to their corresponding receptors on adipocytes leading to altering their homeostasis, rendering them dysfunctional, which decreases the secretion of insulin-sensitizing adipokines and adiponectin, ultimately leading to insulin resistance.^20^

In both clinical practice and our study, there is a clear recognition that the risk of developing T2DM rises with advancing age. This association is thought to arise from a convergence of factors, including age-related declines in pancreatic islet function and an elevated level of insulin resistance.^21^ In our investigation, we used WHR as a marker for insulin resistance. It is also noteworthy that age is a factor known to increase the risk of developing subclinical hypothyroidism, adding complexity to the interplay of age-related metabolic changes.^22^ In fact, the treatment of subclinical hypothyroidism has been a subject of ongoing debate due to several factors, including a natural increase in TSH levels with age, and variability in TSH measurements.^23^ Research findings on this condition have varied, and many studies have suggested associations between subclinical hypothyroidism and various negative health outcomes such as T2DM. Therefore, it is crucial to conduct large randomized controlled trials to definitively establish the clinical impact of levothyroxine therapy across different age groups.

In our study, we identified age as a common cause of both subclinical hypothyroidism and T2DM, making it a confounder in our study (Supplementary Figure 1). To address this confounding effect, we conducted a multivariable logistic regression adjusting for age and other covariates. After running logistic regression diagnostics (Supplementary Figure 2), we found that using age categories as a covariate was superior to utilizing age as a continuous variable. This approach allowed us to account for the confounding effect of age and resulted in a better fit of the data in our model.

Our results can be of clinical relevance since they demonstrate a statistical association between subclinical hypothyroidism and T2DM, which can directly affect patient care in the population, given that further longitudinal studies are conducted to clarify this directionality association.

The sample size of our study was large enough to detect a statistical association between subclinical hypothyroidism and T2DM. Moreover, we adequately adjusted for important covariates in this study by utilizing multivariable logistic regression to adjust for multiple confounders and prognostic variables, which the logistic regression diagnostics confirmed, including the link test and the ROC curve. Our results are important for the public health system in Qatar, warranting robust longitudinal studies. Notably, this study is the first of its kind in Qatar, as there have yet to be any previous reports on the association between hypothyroidism and T2DM in the nation, which could prove useful for further research on the subject.

Our study relied on data from the QBB, which came with a set of limitations. Firstly, due to the way data was collected in the biobank, there is temporal ambiguity. Therefore, further investigations through detailed prospective cohort studies are essential in the backdrop of conflicting results related to the directionality of the association between subclinical hypothyroidism and T2DM.^24^ Additionally, the QBB data only provided single measurements for various biomarkers, including thyroid function tests, for each participant. Therefore, the diagnosis of subclinical hypothyroidism could not be confirmed through the standard requirement of two abnormal thyroid function tests, as is typically done in clinical practice. Furthermore, since the data in our study was anonymized and collected at a single point in time, we were unable to determine the clinical course or the treatment history of the participants in our dataset.

## Conclusion

In the source population of Qatar BioBank, subclinical hypothyroidism was strongly associated with T2DM. However, additional research in this field can aid in better understanding this association.

## References

[1] Williams R, Karuranga S, Malanda B, et al. Global and regional estimates and projections of diabetes-related health expenditure: results from the International Diabetes Federation Diabetes Atlas, 9th edition. *Diabetes Res Clin Pract*. 2020;162:108072. doi:10.1016/j.diabres.2020.108072 32061820

[2] Roden M, Shulman GI. The integrative biology of type 2 diabetes. *Nature*. 2019;576(7785):51–60. doi:10.1038/s41586-019-1797-8 31802013

[3] Halban PA, Polonsky KS, Bowden DW, et al. beta-cell failure in type 2 diabetes: postulated mechanisms and prospects for prevention and treatment. *Diabetes Care*. 2014;37(6):1751–1758. doi:10.2337/dc14-0396 24812433 PMC4179518

[4] Christensen AA, Gannon M. The Beta Cell in Type 2 Diabetes. *Curr Diab Rep*. 2019;19(9):81. doi:10.1007/s11892-019-1196-4 31399863

[5] Sun H, Saeedi P, Karuranga S, et al. IDF Diabetes Atlas: global, regional and country-level diabetes prevalence estimates for 2021 and projections for 2045. *Diabetes Res Clin Pract*. 2022;183:109119. doi:10.1016/j.diabres.2021.109119 34879977 PMC11057359

[6] Ali FM, Nikoloski Z, Reka H, et al. The diabetes-obesity-hypertension nexus in Qatar: evidence from the World Health Survey. *Popul Health Metr*. 2014;12(1):18. doi:10.1186/1478-7954-12-18 25170308 PMC4148083

[7] Awad SF, O’Flaherty M, Critchley J, et al. Forecasting the burden of type 2 diabetes mellitus in Qatar to 2050: a novel modeling approach. *Diabetes Res Clin Pract*. 2018;137:100–108. doi:10.1016/j.diabres.2017.11.015 29175341

[8] Taylor PN, Albrecht D, Scholz A, et al. Global epidemiology of hyperthyroidism and hypothyroidism. *Nat Rev Endocrinol*. 2018;14(5):301–316. doi:10.1038/nrendo.2018.18 29569622

[9] Shaikh S, Honest PC. Burden of hypothyroidism in the primary care population in Qatar. *World Family Medicine*. 2021;19(9):6.

[10] Shahrani A, et al. The epidemiology of thyroid diseases in the Arab world: a systematic review. *J Public Health Epidemiol*. 2016;8(2):10.

[11] Chaker L, Razvi S, Bensenor IM, et al. Hypothyroidism. *Nat Rev Dis Primers*. 2022;8(1):30. doi:10.1038/s41572-022-00357-7 35589725

[12] Hage M, Zantout MS, Azar ST. Thyroid disorders and diabetes mellitus. *J Thyroid Res*. 2011;2011:439463. doi:10.4061/2011/439463 21785689 PMC3139205

[13] Rong F, Dai H, Wu Y, et al. Association between thyroid dysfunction and type 2 diabetes: a meta-analysis of prospective observational studies. *BMC Med*. 2021;19(1):257. doi:10.1186/s12916-021-02121-2 34670571 PMC8529738

[14] Biondi B, Kahaly GJ, Robertson RP. Thyroid Dysfunction and Diabetes Mellitus: two Closely Associated Disorders. *Endocr Rev*. 2019;40(3):789–824. doi:10.1210/er.2018-00163 30649221 PMC6507635

[15] Talwalkar P, Deshmukh V, Bhole M. Prevalence of hypothyroidism in patients with type 2 diabetes mellitus and hypertension in India: a cross-sectional observational study. *Diabetes Metab Syndr Obes*. 2019;12:369–376. doi:10.2147/DMSO.S181470 30936734 PMC6431000

[16] Han C, He X, Xia X, et al. Subclinical Hypothyroidism and Type 2 Diabetes: a Systematic Review and Meta-Analysis. *PLoS One*. 2015;10(8):e0135233. doi:10.1371/journal.pone.0135233 26270348 PMC4535849

[17] Roos A, Bakker SJL, Links TP, et al. Thyroid function is associated with components of the metabolic syndrome in euthyroid subjects. *J Clin Endocrinol Metab*. 2007;92(2):491–496. doi:10.1210/jc.2006-1718 17090642

[18] Mehran L, Amouzegar A, Tohidi M, et al. Serum free thyroxine concentration is associated with metabolic syndrome in euthyroid subjects. *Thyroid*. 2014;24(11):1566–1574. doi:10.1089/thy.2014.0103 25069017

[19] Kalra S, Aggarwal S, Khandelwal D. Thyroid Dysfunction and Type 2 Diabetes Mellitus: screening Strategies and Implications for Management. *Diabetes Ther*. 2019;10(6):2035–2044. doi:10.1007/s13300-019-00700-4 31583645 PMC6848627

[20] Ahirwar AK, Singh A, Jain A, et al. Role of Sub Clinical Hypothyroidism in Association with Adiponectin Levels Causing Insulin Resistance in Metabolic Syndrome: a Case Control Study. *Tokai J Exp Clin Med*. 2017;42(2):96–103. 28681370

[21] De Tata V. Age-related impairment of pancreatic Beta-cell function: pathophysiological and cellular mechanisms. *Front Endocrinol (Lausanne)*. 2014;5:138. doi:10.3389/fendo.2014.00138 25232350 PMC4153315

[22] Leng O, Razvi S. Hypothyroidism in the older population. *Thyroid Res*. 2019;12(1):2. doi:10.1186/s13044-019-0063-3 30774717 PMC6367787

[23] Bekkering GE, Agoritsas T, Lytvyn L, et al. Thyroid hormones treatment for subclinical hypothyroidism: a clinical practice guideline. *BMJ*. 2019;365:l2006. doi:10.1136/bmj.l2006 31088853

[24] Alwan H, Villoz F, Feller M, et al. Subclinical thyroid dysfunction and incident diabetes: a systematic review and an individual participant data analysis of prospective cohort studies. *Eur J Endocrinol*. 2022;187(5):S35–S46. doi:10.1530/EJE-22-0523 36070417 PMC7613845

